# Is *Stomoxys calcitrans* a single species? Morphometric and genetic perspectives from populations in Thailand and Spain

**DOI:** 10.1016/j.crpvbd.2025.100315

**Published:** 2025-09-03

**Authors:** Tanasak Changbunjong, Gérard Duvallet, Sedthapong Laojun, Tanawat Chaiphongpachara

**Affiliations:** a Department of Pre-Clinic and Applied Animal Science, Faculty of Veterinary Science, Mahidol University, Nakhon Pathom, 73170, Thailand; b The Monitoring and Surveillance Center for Zoonotic Diseases in Wildlife and Exotic Animals (MoZWE), Faculty of Veterinary Science, Mahidol University, Nakhon Pathom, 73170, Thailand; c Center d’Ecologie Fonctionnelle et Evolutive (CEFE), Université de Montpellier, Centre National de la Recherche Scientifique (CNRS), École Pratique des Hautes Études (EPHE), Institut de Recherche pour le Développement (IRD), Université Paul Valéry Montpellier 3, 34199, Montpellier, France; d Department of Public Health and Health Promotion, College of Allied Health Sciences, Suan Sunandha Rajabhat University, Samut Songkhram, 75000, Thailand

**Keywords:** Stable fly, Geometric morphometrics, Phylogenetic analysis, Species delimitation, Genetic diversity

## Abstract

*Stomoxys calcitrans* (Diptera: Muscidae) is a significant insect in the veterinary and medical fields. Previous studies have found a pronounced genetic divergence between populations from the Oriental region and those from other zoogeographical zones. Understanding the morphological and genetic variation within *S. calcitrans* populations is essential for clarifying the evolutionary processes underlying their structure across biogeographical regions. This study conducted geometric morphometrics on a total of 120 wings (30 per group: Thailand males, Thailand females, Spain males, and Spain females) to assess wing size and shape variations between *S. calcitrans* populations from Thailand (Oriental region) and Spain (Palaearctic region). Molecular analyses utilized two mitochondrial markers, *cox*1 and *cytb*, the nuclear marker ITS2, and a concatenated dataset of all three. Geometric morphometric analysis revealed statistically significant differences in wing size and shape (*P* < 0.05), although the classification accuracy based on wing shape was moderate (70%), indicating phenotypic plasticity rather than species-level differentiation. Phylogenetic reconstruction identified two well-supported genetic lineages. However, the results from the species delimitation methods (Assemble Species by Automatic Partitioning, Automated Barcode Gap Discovery, and multi-rate Poisson Tree Processes), low interpopulation divergence, and a shared haplotype all verify that these lineages represent a single, globally distributed species. Further neutrality tests and mismatch distribution analyses revealed that the Oriental population has a deeper evolutionary history, while the European population likely arose from a more recent colonization event. These findings demonstrate the influence of historical biogeographical processes in shaping the global genetic structure of *S. calcitrans* and underscore the importance of broader geographical sampling to fully elucidate its evolutionary history.

## Highlights

• Wing shape and size vary between Thai and Spanish *Stomoxys calcitrans* populations.
• Wing differences indicate phenotypic plasticity, not species-level divergence.
• Genetic data revealed two lineages with low divergence and shared haplotypes.
• Species delimitation confirmed that *S. calcitrans* is a single, globally distributed species.
• The Thai population showed a deeper genetic history, whereas that from Spain likely indicates recent colonization.

## Graphical abstract

**Figure:**
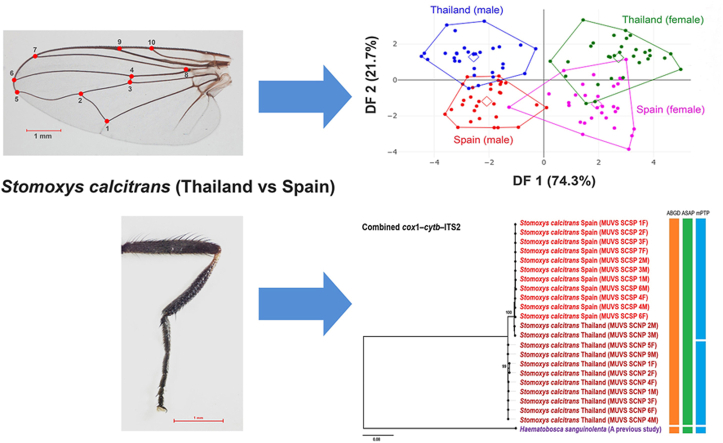

## 1 Introduction

*Stomoxys calcitrans*, commonly known as the stable fly, is a species of the family Muscidae (Order Diptera) with significant veterinary and medical importance (Rochon et al., 2021). Historically, the genus *Stomoxys* was classified within the tribe Stomoxyini of the subfamily Muscinae. However, current taxonomic consensus increasingly substantiates its reassignment to the distinct subfamily Stomoxyinae (Duvallet and Hogsette, 2023). Adult stable flies superficially resemble house flies but are distinguished by a prominent sucking proboscis. Both males and females are obligate blood-feeders that feed on a broad range of domestic and wild animals, particularly cattle. Humans may also be targeted in regions with limited animal hosts (Rochon et al., 2021). The blood-feeding activity of *S. calcitrans* causes substantial irritation, blood loss, and lesion formation. Several studies have established this species as a mechanical vector for various pathogens, including viruses, protozoa, and bacteria (Baldacchino et al., 2013; Hornok et al., 2020). These pathogens are acquired during blood meals from infected hosts and transmitted to other hosts during subsequent feedings. Saliva is injected at the onset of feeding, potentially facilitating the transmission of infectious agents adhering to the fly’s mouthparts (Hornok et al., 2020). *Stomoxys calcitrans* is a significant contributor to economic losses in the livestock industry (Rochon et al., 2021), impairing cattle productivity by causing weight loss and reducing milk yield, primarily due to blood depletion and increased energy expenditure resulting from constant defensive behaviors (Baldacchino et al., 2013). Furthermore, *S. calcitrans* is a transmission vector of several livestock diseases, including equine infectious anemia, lumpy skin disease, besnoitiosis, and trypanosomosis (Foil et al., 1983; Rochon et al., 2021; Makhahlela et al., 2022).

*Stomoxys calcitrans* has become globally distributed and is now found across nearly all continents. Its widespread occurrence presents significant veterinary and medical challenges in many regions (Mavoungou et al., 2008; Baldacchino et al., 2013; Duvallet and Hogsette, 2023). This species is commonly associated with the human environment, thriving in areas where human and animal activities overlap. Notably, it exhibits a high degree of ecological adaptability, successfully persisting across diverse climatic zones, from equatorial regions to high-altitude mountain ranges, and even areas near the poles (Muenworn et al., 2010). Recent technological advancements have significantly improved the study of medically and veterinary important insects. Molecular biology and geometric morphometric (GM) techniques are now widely applied to facilitate species identification while exploring genetic diversity and morphological variation within and between populations (Chaiphongpachara and Laojun, 2019; Changbunjong et al., 2024, 2025).

Although population genetic studies on *S. calcitrans* remain limited, existing molecular data provide meaningful insights into its evolutionary history. Phylogeographical analyses based on mitochondrial and nuclear markers indicate that New World populations likely originated from the Palaearctic region, with their introduction occurring within the past 500 years, potentially facilitated by human activity and livestock movement (Marquez et al., 2007; Dsouli-Aymes et al., 2011). Furthermore, global genetic analyses revealed a pronounced divergence between populations from the Oriental region (encompassing South and Southeast Asia) and those from other major zoogeographical regions. No shared haplotypes were identified between these two lineages, suggesting long-term evolutionary isolation dating back to the mid-Pleistocene (Dsouli-Aymes et al., 2011). This pronounced genetic differentiation could either reflect divergence resulting from historical geographical isolation or indicate the presence of a cryptic species complex within *S. calcitrans*. These possibilities necessitate further investigation through molecular species delimitation methods and the concatenation of multiple genetic loci to produce longer and more informative sequences for robust phylogenetic analysis. In addition, integrating advanced tools such as landmark-based geometric morphometrics, which have proven effective in distinguishing species within the genus *Stomoxys*, may help determine whether the observed genetic lineages represent a single species or distinct taxa.

In this study, the landmark-based GM approach was conducted to assess wing size and shape variation in *S. calcitrans* specimens collected from Thailand (representing the Oriental region) and Spain (representing the Palaearctic region). Furthermore, the mitochondrial markers cytochrome *c* oxidase subunit 1 (*cox*1) and cytochrome *b* (*cytb*), the nuclear marker internal transcribed spacer 2 (ITS2) and a concatenated dataset combining all three, were used to conduct phylogenetic analyses, molecular species delimitation, and genetic diversity and population structure evaluations between the two regions. The results of this integrative approach would improve our understanding of both genetic and morphological differentiation in *S. calcitrans*, as well as elucidate the potential factors driving such variations.

## 2 Materials and methods

### 2.1 Fly specimens

The specimens of *S. calcitrans* were collected in Thailand in 2024 at the equine facility of the Faculty of Veterinary Science, Mahidol University, Nakhon Pathom Province, Thailand (13°47′51′′N, 100°19′07′′E). Five Vavoua insect traps (Laveissière and Grébaut, 1990) were positioned around the stable and operated from 06:00 to 18:00 h. The captured flies were euthanized by freezing at −20 °C and transported to the Vector-Borne Diseases Research Unit within the same faculty for laboratory processing. In the laboratory, the specimens were morphologically identified as *S. calcitrans* using standard taxonomic keys (Zumpt, 1973; Tumrasvin and Shinonaga, 1978) under a Nikon SMZ745 stereomicroscope (Nikon Corp., Tokyo, Japan). The confirmed individuals were placed into separate 1.5-ml microcentrifuge tubes and stored at −20 °C until further analysis. To facilitate interregional comparisons, digital files, including wing images and nucleotide sequence data, obtained from morphologically identified *S. calcitrans* specimens collected in 2024 using Stomoxycc® traps (Alcochem Hygiene, Zeilmaker 4, 3861 SM Nijkerk, The Netherlands) in Bodonal de la Sierra, Badajoz Province, Spain (38°9′25′′N, 6°34′32′′W), a distinct zoogeographical region, were generously provided by Université Paul Valéry Montpellier, France.

### 2.2 Preparing wing images

In total, 60 *S. calcitrans* specimens from Thailand (30 males and 30 females) were selected based on the criterion of having fully intact, undamaged wings. The left wing of each specimen was carefully dissected from the thorax and mounted on a drop of Hoyer’s medium applied to a microscope slide. Using a sterile blade, each wing was gently positioned to ensure it was fully submerged in the medium, after which a coverslip was applied. The prepared slides were left to air-dry for approximately 2–3 days. Upon drying, images of the wing specimens were captured using a digital camera mounted on a Nikon AZ100 stereomicroscope (Nikon Corp.). In total, 60 wing images from *S. calcitrans* specimens from Spain (30 males and 30 females) were also obtained. All images were captured with a scale bar indicating 1 mm.

### 2.3 GM analysis

**Fig. 1:**
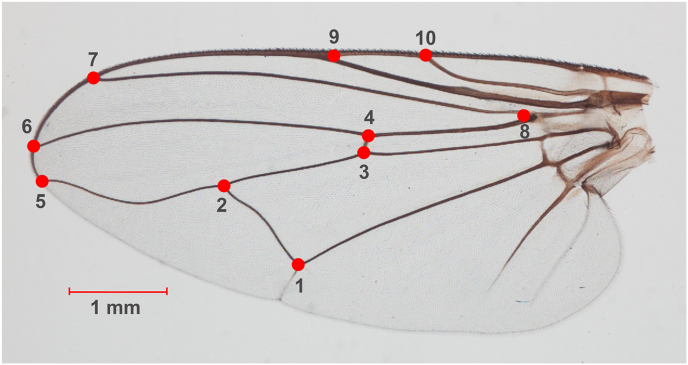
Ten anatomical landmarks located on the left wing of *Stomoxys calcitrans* were used for geometric morphometric analysis. These landmark positions were referenced from previous studies by Changbunjong et al. (2016, 2023) that successfully demonstrated the species-level differentiation of *Stomoxys* in Thailand.

All procedures related to the landmark-based GM approach, including landmark digitization, wing size analysis, wing shape analysis, and validated classification, were conducted using XY Online Morphometrics version 3 (Dujardin and Dujardin, 2019). GM analysis began with the digitization of 10 landmarks positioned at vein intersections and points where wing veins meet the wing margin (Fig. 1). The landmark set employed was based on previous studies that successfully used it for species identification of *Stomoxys* flies in Thailand (Changbunjong et al., 2016, 2023). To assess the precision of landmark digitization, 20 wing images were randomly selected and redigitized by the same individual. The repeatability index (R) was then calculated using Procrustes analysis of variance (Arnqvist and Mårtensson, 1998). The analysis yielded a high repeatability score for shape (99%), indicating that the landmark digitization in this study was both precise and consistent.

For wing size and shape analysis, landmark data were standardized using generalized Procrustes analysis (GPA), which applies Procrustes superimposition to remove variation in size, position, and orientation across all specimens. Wing size was quantified using the centroid size (CS), an isometric measure calculated as the square root of the sum of the squared distances from each landmark to the centroid (geometric center) of the configuration (Bookstein, 1997). To assess significant differences in CS between male and female *S. calcitrans* specimens from Thailand and Spain, non-parametric permutation tests (1000 permutations) with Bonferroni correction were performed. Statistical significance was set at *P* < 0.05. For wing shape analysis, landmark configurations were superimposed to allow visual comparison of the mean shapes. Wing shape variables were derived from GPA and subsequently evaluated by principal components analysis. Sixteen principal components (PCs) were retained and used as the final shape variables in further analyses. Shape variations between male and female *S. calcitrans* specimens from Thailand and Spain were examined using discriminant analysis (DA), with group separation illustrated through factor maps based on the first two discriminant factors (DFs). The Mahalanobis distances were calculated to quantify the shape divergence between the groups. To test the statistical significance of these shape differences, non-parametric permutation (1000 permutations) with Bonferroni correction was applied. Statistical significance was set at *P* < 0.05.

To evaluate the classification accuracy of wing size and shape, a validated classification using the leave-one-out method was performed. This approach assessed the effectiveness of wing size and shape in assigning individuals to their original groups. Classification based on wing size was performed using maximum likelihood (ML) analysis, while classification based on wing shape was based on the Mahalanobis distances. Classification accuracy was reported as the percentage of individuals correctly reassigned to their original groups. To enhance interpretability, the overall accuracy was adjusted based on prior probabilities, resulting in an adjusted total assignment accuracy. This adjustment accounts for the probability of correct classifications occurring by chance, providing a more robust measure of discriminative performance (Klecka, 1980).

The allometric effect, the influence of wing size on wing shape variation, was evaluated by calculating the coefficient of determination (R^2^) from the regression of the first principal component (PC1) of shape on CS. Analyses were conducted for all specimens combined as well as separately for male and female from Thailand and Spain. Statistical significance was estimated using a non-parametric permutation test (1000 permutations), with significance set at *P* < 0.05.

### 2.4 DNA extraction, polymerase chain reaction, and sequencing

Genomic DNA was extracted from two to four legs of each *S. calcitrans* specimen, serving as the tissue source for DNA isolation. Extractions were performed using the DNeasy® Blood & Tissue Kit (Qiagen, Hilden, Germany) according to the manufacturer’s standard protocol. For molecular characterization, three genetic markers were selected based on their proven effectiveness in a previous study on the global genetic structure of *S. calcitrans* (Dsouli-Aymes et al., 2011). These included the two mitochondrial genes *cox*1 and *cytb* and the nuclear marker ITS2. Each target region was amplified by polymerase chain reaction (PCR) using specific primer pairs: *cox*1 (658 bp) was amplified using LepF1 (5′-ATT CAA CCA ATC ATA AAG ATA TTG G-3′) and LepR1 (5′-TAA ACT TCT GGA TGT CCA AAA AAT CA-3′) (Hebert et al., 2004); *cytb* (592 bp) using CB-J10933 (5′-GTT TTA CCT TGA GGA CAA ATA TC-3′) and CB-N11526 (5′-TTC AAC TGG TCG AGC TCC AAT TCA-3′) (Simon et al., 1994); and ITS2 (∼360 bp) using ITS2A (5′-TGT GAA CTG CAG GAC ACA T-3′) and ITS2-F (5′-TAT GCT TAA ATT CAG GGG GT-3′) (Simon et al., 1994).

PCRs for amplifying *cox*1, *cytb*, and ITS2 were performed in 25-μl reaction volumes, each consisting of 1× PCR buffer, 1.5 mM MgCl_2_, 0.2 mM deoxyribonucleotide triphosphate, 1.5 units of Platinum Taq DNA Polymerase (Invitrogen, Carlsbad, USA), 0.3 μM of each primer, 5 μl DNA template, and nuclease-free water (ddH_2_O) to complete the final volume. The thermal cycling conditions for *cox*1 amplification began with initial denaturation at 94 °C for 1 min, followed by 5 cycles of denaturation at 94 °C for 30 s, annealing at 45 °C for 40 s, and extension at 72 °C for 1 min. This was followed by 35 additional cycles at 94 °C for 30 s, 55 °C for 40 s, and 72 °C for 1 min. A final extension was performed at 72 °C for 10 min, followed by a hold at 4 °C. For *cytb* and ITS2, the PCR profile included an initial denaturation at 94 °C for 4 min, followed by 35 cycles of 94 °C for 40 s, annealing at 58 °C (*cytb*) or 61 °C (ITS2) for 40 s, and extension at 72 °C for 1 min. This was followed by a final extension at 72 °C for 10 min and a subsequent hold at 4 °C. Each PCR run incorporated both positive and negative controls to ensure amplification accuracy and prevent false results. The positive control consisted of *S. calcitrans* DNA obtained from a previously validated specimen (Mongkolphan et al., 2023), confirming the efficacy of primer annealing and optimal reaction conditions. The negative control, prepared without the template DNA, was used to detect any possible contamination. Following amplification, the PCR products were separated by electrophoresis on 1.5% agarose gels and visualized under ultraviolet (UV) light using a gel documentation system (GE Healthcare Japan Corporation, Tokyo, Japan). This step verified amplification success by confirming both the expected amplicon size and band intensity. PCR products with satisfactory quality were then sent to Solgent Co., Ltd. (Daejeon, South Korea) for purification and DNA sequencing.

### 2.5 Phylogenetic analysis and species delimitation

Molecular analyses were conducted using DNA sequence data from 24 *S. calcitrans* specimens, 12 from Thailand and 12 from Spain (Table 1). The bidirectional Sanger chromatograms obtained for *cox*1, *cytb*, and ITS2 were manually examined to ensure sequence integrity. Low-quality regions at the 5′- and 3′-ends of both forward and reverse reads were trimmed, and any artifactual polymorphisms, such as ambiguous base calls or sequencing artifacts, were corrected. The forward and reverse reads were then aligned and merged to generate high-quality consensus sequences using BioEdit software version 7.2.5 (Hall, 1999). To reconfirm species identity, all consensus sequences were queried against the NCBI nucleotide database using BLAST (Basic Local Alignment Search Tool), allowing confirmation through sequence homology (Altschul et al., 1990). The finalized sequences were subsequently deposited in GenBank, and their respective accession numbers are provided in Table 1.

**Table 1:** Voucher specimens with GenBank accession numbers for the mitochondrial (*cox*1 and *cytb*) and nuclear (ITS2) sequences of *Stomoxys calcitrans* specimens collected in Spain and Thailand.

Specimen-voucher	Geographical location	Sex	GenBank accession number		
			*cox*1	*cytb*	ITS2
MUVS:SCSP:1F	Spain	Female	PV750979	PV759149	PV754105
MUVS:SCSP:2F	Spain	Female	PV750980	PV759150	PV754106
MUVS:SCSP:3F	Spain	Female	PV750981	PV759151	PV754107
MUVS:SCSP:4F	Spain	Female	PV750982	PV759152	PV754108
MUVS:SCSP:6F	Spain	Female	PV750983	PV759153	PV754109
MUVS:SCSP:7F	Spain	Female	PV750984	PV759154	PV754110
MUVS:SCSP:1M	Spain	Male	PV750985	PV759155	PV754111
MUVS:SCSP:2M	Spain	Male	PV750986	PV759156	PV754112
MUVS:SCSP:3M	Spain	Male	PV750987	PV759157	PV754113
MUVS:SCSP:4M	Spain	Male	PV750988	PV759158	PV754114
MUVS:SCSP:5M	Spain	Male	PV750989	PV759159	NS
MUVS:SCSP:6M	Spain	Male	PV750990	PV759160	PV754115
MUVS:SCNP:1F	Thailand	Female	PV750991	PV759161	PV754116
MUVS:SCNP:2F	Thailand	Female	PV750992	PV759162	PV754117
MUVS:SCNP:3F	Thailand	Female	PV750993	PV759163	PV754118
MUVS:SCNP:4F	Thailand	Female	PV750994	PV759164	PV754119
MUVS:SCNP:5F	Thailand	Female	PV750995	PV759165	PV754120
MUVS:SCNP:6F	Thailand	Female	PV750996	PV759166	PV754121
MUVS:SCNP:1M	Thailand	Male	PV750997	PV759167	PV754122
MUVS:SCNP:2M	Thailand	Male	PV750998	PV759168	PV754123
MUVS:SCNP:3M	Thailand	Male	PV750999	PV759169	PV754124
MUVS:SCNP:4M	Thailand	Male	PV751000	PV759170	PV754125
MUVS:SCNP:9M	Thailand	Male	PV751001	PV759171	PV754126
MUVS:SCNP:10M	Thailand	Male	PV751002	PV759172	NS

*Abbreviation*: NS, PCR amplification was unsuccessful.

Genetic distances were calculated using the Kimura 2-Parameter (K2P) model to assess intra- and inter-group divergences for the *cox*1, *cytb*, ITS2, and combined *cox*1-*cytb*-ITS2 sequences using MEGA 11 software (Tamura et al., 2021). Sequence alignment was conducted using ClustalW (Larkin et al., 2007), after which ML phylogenetic analyses were performed to infer the genetic relationships among the sampled populations. Separate ML trees were constructed for each marker, as well as for the combined dataset (*cox*1-*cytb*-ITS2), incorporating reference sequences from various Asian and European countries retrieved from GenBank. Phylogenetic trees were rooted using additional *Stomoxys* species and an external outgroup, *Haematobosca sanguinolenta*, a closely related taxon outside the genus. All phylogenetic analyses were conducted using MEGA 11. Prior to analyses, the best-fitting nucleotide substitution models were selected based on the lowest Bayesian Information Criterion values. The general time reversible model with a gamma distribution (GTR+G) was selected for the *cox*1 dataset, while the general time reversible model with a proportion of invariant sites (GTR+I) was identified as optimal for *cytb*. The ITS2 dataset was best described by the Tamura 3-parameter model with a gamma distribution (T92+G). For the combined *cox*1-*cytb*-ITS2 sequences, the GTR+I model provided the best fit.

DNA-based species delimitation analyses were conducted on the mitochondrial (*cox*1 and *cytb*), nuclear (ITS2), and combined (*cox*1-*cytb*-ITS2) sequences. In this study, three complementary approaches were employed: Assemble Species by Automatic Partitioning (ASAP) (Puillandre et al., 2021), Automated Barcode Gap Discovery (ABGD) (Puillandre et al., 2021), and multi-rate Poisson Tree Processes (mPTP) (Kapli et al., 2017). ASAP and ABGD are distance-based methods that detect the presence of a “barcode gap” to differentiate between intraspecific variation and interspecific divergence, thereby clustering sequences into provisional Molecular Operational Taxonomic Units (MOTU). ABGD (https://bioinfo.mnhn.fr/abi/public/abgd/abgdweb.html) employs a recursive partitioning algorithm based on pairwise genetic distance distributions, whereas ASAP (https://bioinfo.mnhn.fr/abi/public/asap/) uses hierarchical clustering and ranks partitions based on a scoring system that incorporates barcode gap width and the probability of panmixia. Both analyses were performed online under default settings using p-distance metrics (Krčmar et al., 2022). In contrast, the mPTP method is a tree-based, phylogeny-aware approach for species delimitation, also with a web-based platform (https://mptp.h-its.org/#/tree). It identifies putative species boundaries by analyzing the distribution of branch lengths within a phylogenetic tree to clarify variable substitution rates across lineages. Unlike the distance-based methods, mPTP does not rely on predefined threshold values but instead detects shifts in the branching patterns indicative of speciation events.

### 2.6 Genetic diversity and haplotype analysis

Genetic diversity indices for *S. calcitrans* populations sampled in Thailand and Spain were also determined using the mitochondrial (*cox*1, *cytb*), nuclear (ITS2), and combined (*cox*1-*cytb*-ITS2) sequences. The calculated parameters included the number of sequences analyzed (*n*), number of polymorphic or segregating sites (*s*), average number of nucleotide differences (*κ*), nucleotide diversity (*π*), number of haplotypes (*h*), and haplotype diversity (*Hd*). All analyses were performed using DnaSP version 6.12.03 (Rozas et al., 2017). To explore the genetic relationships among the haplotypes, median-joining haplotype networks were constructed for each marker using PopART version 1.7 (Leigh and Bryant, 2015). These networks incorporated the sequences generated in this study, along with several reference sequences from other Asian and European regions obtained from GenBank.

### 2.7 Population genetic analysis

To assess the genetic structure of the *S. calcitrans* populations, analysis of molecular variance (AMOVA) was conducted using pairwise nucleotide differences on ARLEQUIN version 3.5.2.2 (Excoffier and Lischer, 2010). The significance of the test statistics was evaluated through 1000 permutations. This analysis partitioned the total genetic variation into components attributable to inter- and intra-population differences. Genetic differentiation between the Thai and Spanish populations was quantified using *F*_*ST*_ (fixation index) values, which indicate population divergence. These values provided insights into the degree of geographical isolation and potential evolutionary differentiation between the Thai and Spanish populations.

To test for population equilibrium, neutrality tests (Fu’s *F*_*s*_ and Tajima’s *D*) were performed using DnaSP to identify signals of population expansion or selection. To further assess whether the two populations had undergone sudden demographic expansion, mismatch distribution analysis based on combined *cox*1-*cytb*-ITS2 sequences was performed in ARLEQUIN. This method evaluates the distribution of pairwise nucleotide differences among sequences to determine the patterns of recent expansion. Under the sudden expansion model, two key parameters, the sum of squared deviations (SSD) and Harpending’s raggedness index (Rg), were used to assess deviations from the model’s expected distribution.

## 3 Results

### 3.1 GM analysis of wing size

**Fig. 2:**
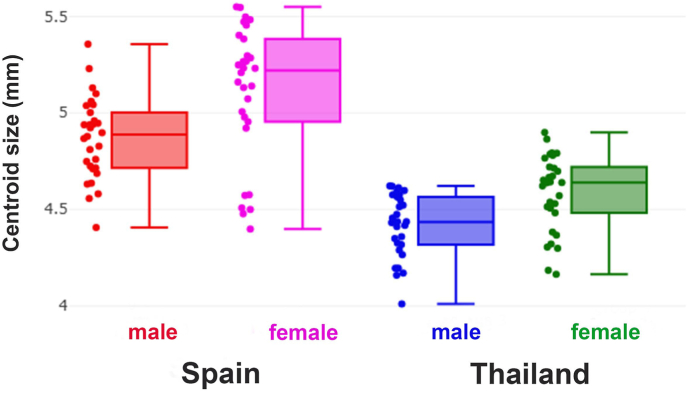
Boxplots illustrating wing centroid size variations among the four groups of male and female *Stomoxys calcitrans* specimens collected from Thailand and Spain. Each box represents the interquartile range (25th–75th percentiles), with the central line indicating the median. The individual data points beside each box indicate the size of individual specimens (in mm).

The wing CS ranged from 4.01 to 5.55 mm (Table 2, see boxplots presented in Fig. 2). Fig. 2 illustrates intergroup variation in wing CS, showing clear patterns of geographical differentiation. The highest mean CS was recorded in Spanish females (5.11 mm), followed by Spanish males (4.87 mm), Thai females (4.58 mm), and Thai males (4.42 mm). The differences among all groups were significant (*P* < 0.05; Table 2). The validated classification based solely on wing size yielded a relatively low adjusted total accuracy of 41% (Table 3).

**Table 2:** Wing centroid size measurements among the four sex-location groups of *Stomoxys calcitrans* specimens collected in Thailand and Spain.

Group	*n*	Mean (mm)	Min-Max (mm)	Variance	SD
Spain (♂)	30	4.87^A^	4.41–5.36	0.04	0.21
Spain (♀)	30	5.11^B^	4.40–5.55	0.12	0.35
Thailand (♂)	30	4.42^C^	4.01–4.62	0.03	0.16
Thailand (♀)	30	4.58^D^	4.16–4.90	0.04	0.20

*Note*: Different superscript letters indicate statistically significant differences among the groups (*P* < 0.05).

*Abbreviation*: SD, standard deviation; Min, minimum; Max, maximum.

**Table 3:** Validated classification accuracy based on wing size and wing shape for the four sex-location groups of *Stomoxys calcitrans* specimens collected in Thailand and Spain.

Group	Wing size		Wing shape	
	Accuracy (%)	Assigned/Observed	Accuracy (%)	Assigned/Observed
Spain (♂)	43.33	13/30	80.00	24/30
Spain (♀)	73.33	22/30	66.67	20/30
Thailand (♂)	60.00	18/30	76.67	23/30
Thailand (♀)	46.67	14/30	86.67	26/30
Total accuracy	55.83	67/120	77.50	93/120
Adjusted total accuracy	41.00		70.00	

*Notes*: Accuracy is expressed as the percentage of correctly classified specimens. “Assigned/Observed” indicates the number of specimens correctly assigned to their original group relative to the total observed. “Adjusted total accuracy” reflects chance-corrected classification performance by minimizing the influence of random variation.

### 3.2 GM analysis of wing shape

**Fig. 3:**
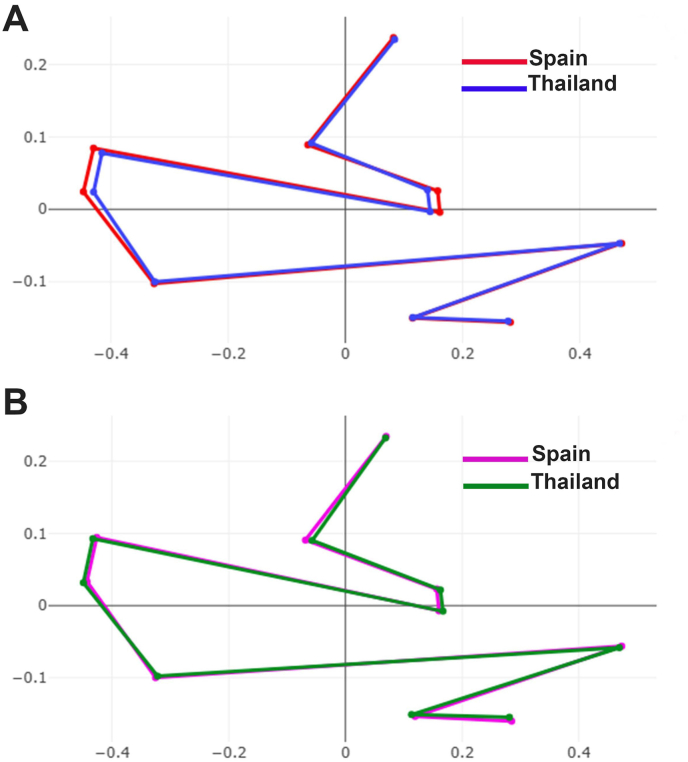
Superimposed mean landmark configurations of male (**A**) and female (**B**) *Stomoxys calcitrans* specimens collected from Thailand and Spain, highlighting initial differences in wing shape between populations from the two regions.

**Fig. 4:**
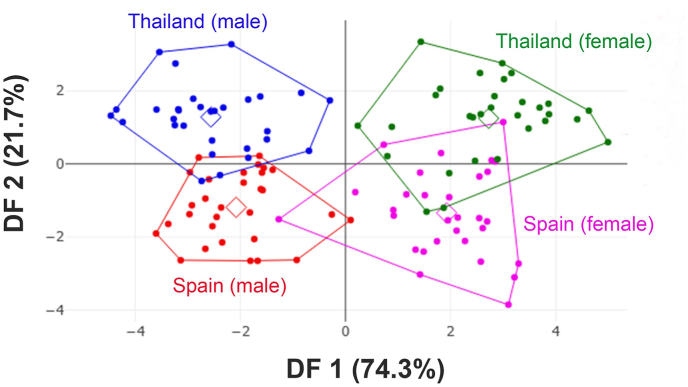
Discriminant factor (DF) map derived from the final wing shape variables of male and female *Stomoxys calcitrans* specimens collected from Thailand and Spain. Each point represents an individual specimen. The horizontal and vertical axes correspond to the first and second DFs (DF1 and DF2), respectively.

The superimposed mean shapes based on those derived from landmark configurations revealed distinct wing shape differences between the two *S. calcitrans* populations at certain landmark coordinates (Fig. 3). Divergence in wing shape was more pronounced between males from the two populations than between females. DA was performed to further assess group differentiation. The resulting factor map (Fig. 4), constructed from two discriminant functions (DF1 and DF2), accounted for 96% of the total shape variation, 74.3% from DF1 and 21.7% from DF2. Although the scatterplot showed some degree of group separation, partial overlap remained evident.

Mahalanobis distances calculated to quantify the shape differences among the four subgroups showed values ranging from 2.76 to 5.39 (Table 4). All pairwise comparisons were statistically significant (*P* < 0.05), with the highest distance observed between Thai females and Spanish males, reflecting shape divergence influenced by both sex and geography. The validated classification based on wing shape achieved an adjusted total accuracy of 70%, indicating a moderate discriminatory potential for distinguishing between the two populations.

**Table 4:** Mahalanobis distances among the four sex-location groups of *Stomoxys calcitrans* specimens collected in Thailand and Spain.

Group	Spain (♂)	Spain (♀)	Thailand (♂)
Spain (♂)	–		
Spain (♀)	4.19∗	–	
Thailand (♂)	2.76∗	5.20∗	–
Thailand (♀)	5.39∗	2.91∗	5.37∗

*Note*: ∗*P* < 0.05.

### 3.3 Allometry

The allometric effect of wing size on wing shape was significant in *S. calcitrans* when considering all specimens combined (r = 0.306, R^2^ = 9.4%; *P* < 0.05). When analyzed separately, male specimens from Thailand and Spain showed r = –0.257 (R^2^ = 6.6%; *P* < 0.05), and female specimens from Thailand and Spain showed r = –0.282 (R^2^ = 7.9%; *P* < 0.05).

### 3.4 Intra- and interspecific genetic variation

Genetic divergence analysis based on the K2P model revealed distinct patterns of intra- and inter-group variation between the populations of *S. calcitrans* from Thailand and Spain using mitochondrial (*cox*1 and *cytb*), nuclear (ITS2), and combined sequence data (Table 5).

**Table 5:** Intra- and inter-population genetic divergences for the *cox*1 (658 bp), *cytb* (592 bp), ITS2 (∼360 bp), and combined *cox*1-*cytb*-ITS2 (∼1610 bp) sequences between *Stomoxys calcitrans* populations from Thailand and Spain.

Genetic marker	Intra-population divergence (%)		Inter-population divergence (%)
	Thailand (Min-Max)	Spain (Min-Max)	Thailand *vs* Spain (Min-Max)
*cox*1	0.82 (0.00–2.32)	0.14 (0.00–0.46)	1.99 (0.30–2.64)
*cytb*	0.55 (0.00–1.72)	0.08 (0.00–0.17)	1.51 (0.68–1.89)
ITS2	0.10 (0.00–0.56)	0.00 (0.00–0.00)	0.51 (0.00–0.56)
Combined *cox*1*-cytb*-ITS2	0.60 (0.00–1.71)	0.09 (0.00–0.25)	1.47 (0.37–1.90)

*Note*: Values were calculated using the Kimura 2-parameter (K2P) model.

Interpopulation divergence consistently exceeded intrapopulation levels, with the mitochondrial markers exhibiting the highest degrees of genetic differentiation between the two populations. Specifically, *cox*1 showed an interpopulation divergence of 1.99% (range: 0.30–2.64%), while *cytb* exhibited 1.51% (range: 0.68–1.89%), both substantially exceeding their respective intrapopulation values, thereby reflecting a clear pattern of geographical genetic structuring. Moreover, the Thai population exhibited consistently greater intrapopulation genetic variation than the Spanish population across all the analyzed markers, indicating higher genetic heterogeneity within the Thai population.

### 3.5 Phylogenetic tree and species delimitation

**Fig. 5:**
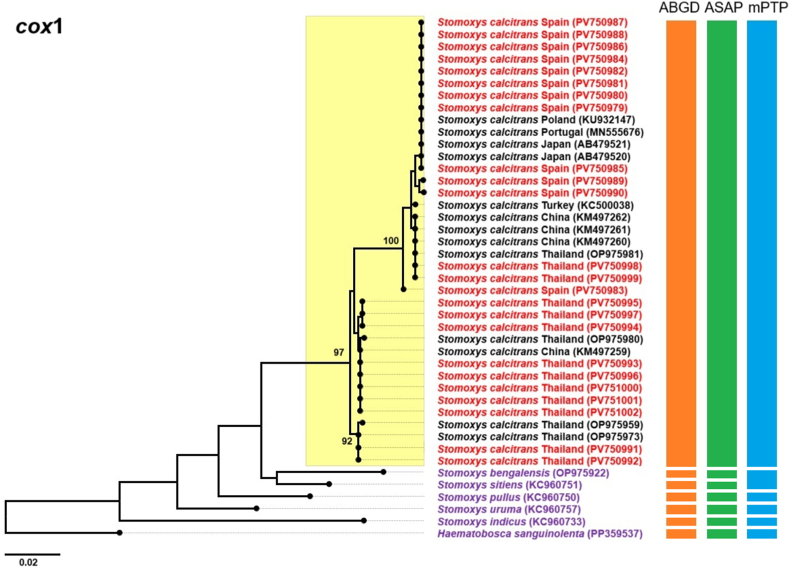
Maximum likelihood phylogenetic tree inferred using the general time reversible model with a Gamma distribution (GTR+G) based on the *cox*1 gene sequences of *Stomoxys calcitrans* specimens from this study (Thailand and Spain; shown in *red*), along with reference sequences from Asian (China, Thailand, and Japan) and European (Poland, Portugal, and Turkey) countries retrieved from the GenBank database (shown in *black*). The outgroup taxa include five additional *Stomoxys* species (*S. bengalensis*, *S. sitiens*, *S. pullus*, *S. uruma*, and *S. indicus*) and *Haematobosca sanguinolenta* (shown in *purple*), which lies outside the genus. Bootstrap support values ≥ 90% are indicated at the corresponding nodes. Color-coded bars represent operational taxonomic units (OTUs) inferred from three species delimitation methods: Automated Barcode Gap Discovery (ABGD; *orange*), Automatic Species Partitioning (ASAP; *green*), and multi-rate Poisson Tree Processes (mPTP; *blue*).

**Fig. 6:**
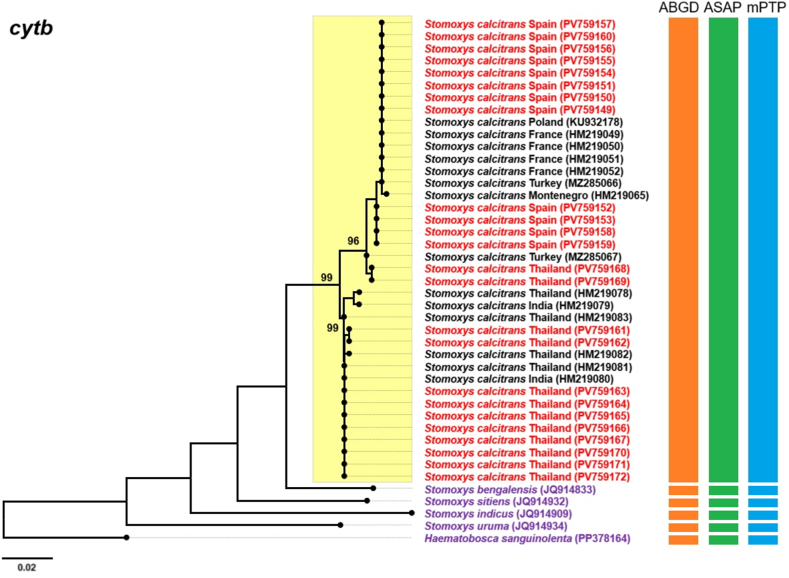
Maximum likelihood phylogenetic tree inferred using the general time reversible model with an invariant site distribution (GTR+I) based on *cytb* gene sequences of *Stomoxys calcitrans* specimens from this study (Thailand and Spain; shown in *red*), along with reference sequences from Asian (Thailand and India) and European (Montenegro, Poland, France, and Turkey) countries retrieved from the GenBank database (shown in *black*). The outgroup taxa include four additional *Stomoxys* species (*S. bengalensis*, *S. sitiens*, *S. indicus*, and *S. uruma*) and *Haematobosca sanguinolenta* (shown in *purple*), which lies outside the genus. Bootstrap support values ≥ 90% are indicated at the corresponding nodes. Color-coded bars represent operational taxonomic units inferred from three species delimitation methods: Automated Barcode Gap Discovery (ABGD; *orange*), Automatic Species Partitioning (ASAP; *green*), and multi-rate Poisson Tree Processes (mPTP; *blue*).

**Fig. 7:**
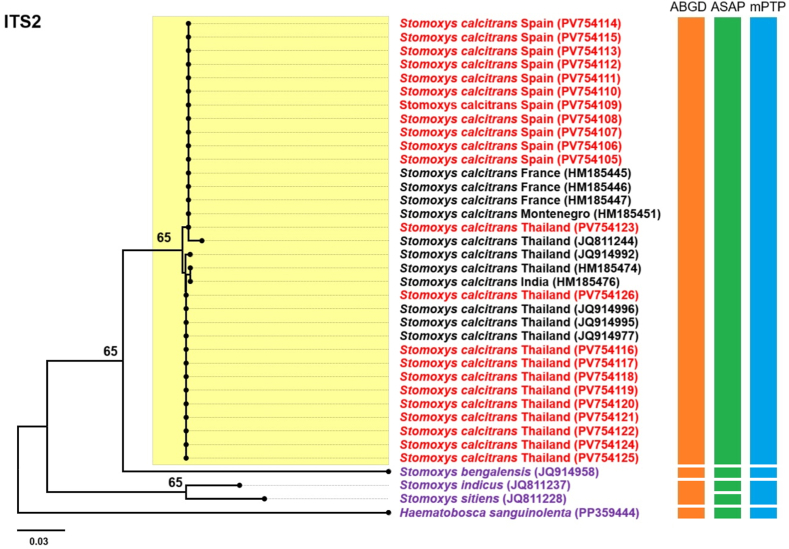
Maximum likelihood phylogenetic tree inferred using the Tamura 3-parameter model with Gamma distribution (T92+G), based on ITS2 sequences of *Stomoxys calcitrans* specimens from this study (Thailand and Spain; shown in *red*), along with reference sequences from Asian (Thailand and India) and European (Montenegro and France) countries retrieved from the GenBank database (shown in *black*). The outgroup taxa include three additional *Stomoxys* species (*S. bengalensis*, *S. indicus*, and *S. sitiens*) and *Haematobosca sanguinolenta* (shown in *purple*), which lies outside the genus. Bootstrap support values ≥ 65% are indicated at the corresponding nodes. Color-coded bars represent operational taxonomic units inferred from three species delimitation methods: Automated Barcode Gap Discovery (ABGD; *orange*), Automatic Species Partitioning (ASAP; *green*), and multi-rate Poisson Tree Processes (mPTP; *blue*).

**Fig. 8:**
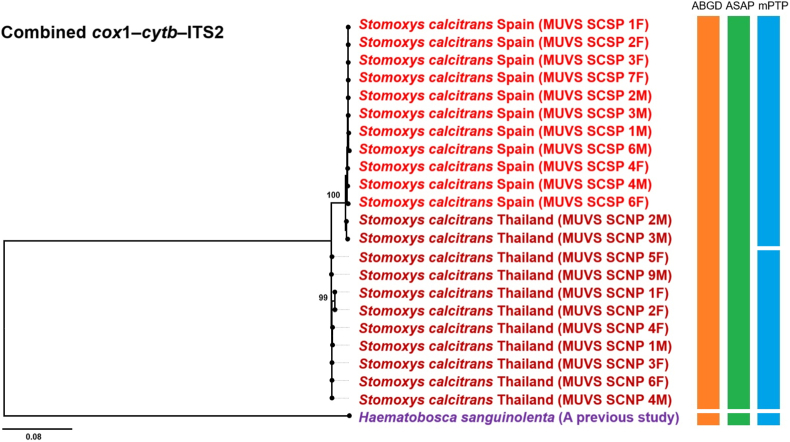
Maximum likelihood phylogenetic tree inferred using the general time reversible model with a proportion of invariant sites (GTR + I), based on the combined *cox*1-*cytb*-ITS2 sequences of *Stomoxys calcitrans* specimens in the present study (Thailand and Spain; shown in *red*). The outgroup taxon, *Haematobosca sanguinolenta* (shown in purple), was adopted based on a previous study. Bootstrap support values ≥ 90% are indicated at the corresponding nodes. Color-coded bars represent operational taxonomic units inferred from three species delimitation methods: Automated Barcode Gap Discovery (ABGD; *orange*), Automatic Species Partitioning (ASAP; *green*), and multi-rate Poisson Tree Processes (mPTP; *blue*).

ML phylogenetic trees based on *cox*1, *cytb*, ITS2, and combined sequence data yielded similar overall topologies (Fig. 5, Fig. 6, Fig. 7, Fig. 8). The *cox*1-based tree revealed that all *S. calcitrans* sequences from both regions formed a well-supported monophyletic clade. Within this clade, two distinct subclades were observed: Subclade 1, representing a predominantly European lineage, and Subclade 2, representing a predominantly Thai lineage (Fig. 5). Subclade 1 was mostly composed of sequences from Spain, along with sequences from other European countries, including Portugal, Poland, and Turkey, as well as two sequences from Japan (GenBank: AB479521 and AB479520), three from China (GenBank: KM497260, KM497261, and KM497262), and three from Thailand (GenBank: OP975981, PV750998, and PV750999), demonstrating genetic overlap across geographical regions. In contrast, Subclade 2 consisted almost entirely of Thai sequences, as well as a single Chinese sequence (GenBank: KM497260), but no other sequences from other countries. All outgroup sequences were clearly separated from the *S. calcitrans* clade. The other *Stomoxys* species branched successively outside the *S. calcitrans* clade. *Haematobosca sanguinolenta*, as a species from a related but distinct genus, was positioned furthest from the *Stomoxys* species, appropriately serving as a distant outgroup.

Phylogenetic trees constructed from *cytb* and ITS2 sequences displayed topologies largely consistent with the *cox*1-based tree, each revealing two distinct subclades within the *S. calcitrans* clade. In the *cytb*-based tree (Fig. 6), the majority of sequences in Subclade 1, were from European countries, including Spain, Poland, France, Turkey, and Montenegro, along with two Thai sequences (GenBank: PV759168 and PV759169). Subclade 2 consisted primarily of Thai sequences and included one Indian sequence from GenBank (GenBank: HM219080) but no European sequences. The ITS2-based tree (Fig. 7) showed a similar composition. Subclade 1 included most European sequences, specifically from Spain, France, and Montenegro, along with two Thai sequences (GenBank: PV759123 and JQ811244). Subclade 2 was composed predominantly of Thai specimens and included a single Indian sequence (GenBank: HM185476) but no European sequences. The phylogenetic tree based on the combined dataset (Fig. 8) revealed two well-supported subclades: one containing Spanish sequences and two Thai sequences (vouchers MUVS:SCNP:2M and MUVS:SCNP:3M), and a second composed exclusively of Thai sequences.

Additional phylogenetic analyses were performed using *S. calcitrans* sequences from this study (Thailand and Spain; PV759149-PV759172 for *cytb* and PV754105-PV754126 for ITS2) together with reference sequences from six zoogeographical regions, Oriental, Afrotropical, Palaearctic, Nearctic, Neotropical, and Oceania. The resulting phylogenies based on both the *cytb* gene and the ITS2 region showed grouping consistent with the grouping of European and Thai populations (Supplementary Figs. 1 and 2). Analyses of the *cox*1 gene were not conducted, as available *S. calcitrans* sequences from other regions were generated using primers targeting different positions than those employed in this study.

Species delimitation was conducted using ASAP, ABGD, and mPTP on the *cox*1, *cytb*, ITS2, and combined (*cox*1-*cytb*-ITS2) sequences. In the ASAP analysis, the optimal partition scores were 5.0 for *cox*1, 3.5 for *cytb*, 4.5 for ITS2, and 5.0 for the combined dataset. ABGD identified partitions using prior maximal intraspecific divergence thresholds of 0.022 for *cox*1, 0.013 for *cytb*, 0.008 for ITS2, and 0.022 for the combined sequences. The mPTP method produced the best multi-rate coalescent scores of 119.78 for *cox*1, 73.08 for *cytb*, 44.62 for ITS2, and 69.71 for the combined dataset. All three methods identified a single common operational taxonomic unit (OTU) encompassing all *S*. *calcitrans* sequences from both this study and GenBank. The mPTP analysis of the combined dataset delineated two putative groups within *S. calcitrans*, consistent with geographical structuring, but the overall pattern across methods corresponded to a single species (Fig. 5, Fig. 6, Fig. 7, Fig. 8).

### 3.6 Genetic diversity

Genetic diversity analysis consistently revealed greater variation across all examined genetic markers in *S*. *calcitrans* specimens from Thailand than those from Spain (Table 6). For both *cox*1 and *cytb*, the Thai population exhibited markedly higher nucleotide diversity (*π* = 0.008 and 0.006, respectively) and haplotype diversity (*Hd* = 0.773 and 0.727), while lower values were obtained for the Spanish population (*π* = 0.001 for both; *Hd* = 0.455 and 0.485). Overall, the ITS2 marker showed low genetic variability, with the Thai population showing only minimal diversity (*π* = 0.001 *Hd* = 0.182) and the Spanish population exhibited none. Analysis of the combined dataset (*cox*1-*cytb*-ITS2) offered the highest genetic resolution. The Thai population showed greater haplotype richness (7 haplotypes), higher diversity indices (*π* = 0.006, *Hd* = 0.909), and a larger average number of nucleotide differences (*κ* = 9.564) than the Spanish population.

**Table 6:** Genetic diversity indices for the *cox*1, *cytb*, ITS2, and combined *cox*1-*cytb*-ITS2 sequences from *Stomoxys calcitrans* populations collected in Thailand and Spain.

Genetic marker	Group	*n*	*h*	*s*	*π* ± SD	*Hd* ± SD	*κ*	*Neutrality test*	
								*Tajima*’*s D*	*Fu*’*s F*_*s*_
*cox*1	Thailand	12	4	16	0.008 ± 0.003	0.773 ± 0.083	5.348	−0.217	3.907
	Spain	12	4	4	0.001 ± 0.001	0.455 ± 0.170	0.939	−1.023	−0.753
*cytb*	Thailand	12	4	10	0.006 ± 0.002	0.727 ± 0.109	3.212	−0.123	2.180
	Spain	12	2	1	0.001 ± 0.000	0.485 ± 0.106	0.485	1.066	1.003
ITS2	Thailand	11	2	2	0.001 ± 0.001	0.182 ± 0.144	0.364	−1.430	0.506
	Spain	11	1	0	0.000 ± 0.000	0.000 ± 0.000	0.000	–	–
Combined *cox*1-*cytb*-ITS2	Thailand	11	7	28	0.006 ± 0.002	0.909 ± 0.066	9.564	−0.158	1.269
	Spain	11	5	5	0.001 ± 0.000	0.709 ± 0.137	1.455	−0.567	−1.143

*Note*: No significant results were obtained for Tajima’s *D* or Fu’s *F*_*s*_ in any of the populations.

*Abbreviations*: *n*, number of sequences analyzed; *h*, number of haplotypes; *s*, number of polymorphic (segregating) sites; *π*, nucleotide diversity; *Hd*, haplotype diversity; *κ*, average number of nucleotide differences.

### 3.7 Haplotype diversity

**Fig. 9:**
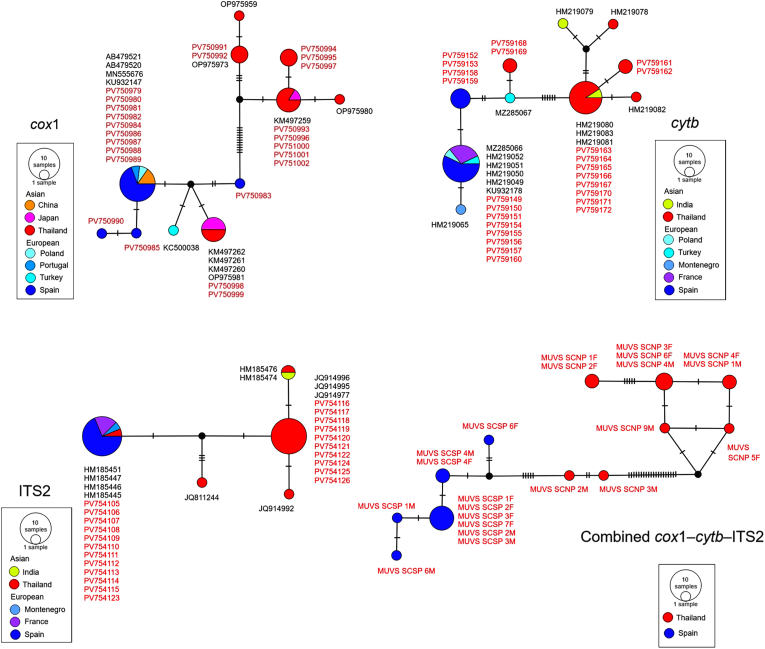
Median-joining haplotype networks of *Stomoxys calcitrans*, constructed using mitochondrial markers (*cox*1 and *cytb*), a nuclear marker (ITS2), and their concatenated sequences (combined *cox*1-*cytb*-ITS2). The networks include sequences generated in this study from specimens collected in Thailand and Spain (indicated in *red*), along with reference sequences from various Asian and European countries retrieved from the GenBank database (shown in *black*). Each circle represents a unique haplotype, with its size proportional to the number of individuals sharing that haplotype. Phylogroups based on geographical origin are indicated with different colors. The lines connecting circles represent mutational steps, with hatch marks indicating the number of base pair differences. Black dots denote unsampled or missing intermediate haplotypes.

Median-joining haplotype network analyses based on the *cox*1, *cytb*, ITS2, and combined *cox*1-*cytb-*ITS2 sequences illustrated the genetic relationships among *S. calcitrans* populations from Thailand and Spain (Fig. 9). To clarify these relationships, a diverse set of representative sequences from Asia and Europe retrieved from the GenBank database was incorporated. These reference sequences were identical to those used in the phylogenetic tree constructions, ensuring consistency across analyses. The haplotype networks revealed partial geographical structuring, with multiple mutational steps generally separating the haplotypes between the two regions. Some Asian sequences were positioned within clusters predominantly composed of European samples.

Furthermore, haplotype network analyses of *cox*1 and *cytb* showed no shared haplotypes between the Thai and Spanish populations, with each forming distinct and mutually exclusive haplotype clusters. In contrast, the network based on ITS2 included one shared haplotype between populations from both regions. For the network based on combined *cox*1-*cytb*-ITS2, the haplotypes were entirely segregated by geographical origin, with the Thai and Spanish sequences forming clearly separated, non-overlapping clusters.

### 3.8 Population structure and genetic differentiation

The AMOVA results for the *cox*1, *cytb*, ITS2, and combined *cox*1-*cytb*-ITS2 datasets revealed pronounced and statistically significant genetic structuring between the two *S. calcitrans* populations (Table 7). For all genetic markers, most genetic variation was attributed to differences among populations rather than within populations. Furthermore, all markers yielded significantly high *F*_*ST*_ values: 0.755 for *cox*1; 0.791 for *cytb*; 0.900 for ITS2; and 0.758 for the combined dataset (*P* < 0.001 for all comparisons; Table 7).

**Table 7:** Analysis of molecular variance based on *cox*1, *cytb*, ITS2, and combined *cox*1-*cytb*-ITS2 sequences from *Stomoxys calcitrans* populations collected in Thailand and Spain.

Genetic marker	Source of variation	Sum of squares	Variance component	% Variation	*F*_*ST*_	*P*-value
*cox*1	Among populations	59.792	4.852	75.53	0.755	<0.001
	Within populations	34.583	1.572	24.47		
	Total	94.375	6.424			
*cytb*	Among populations	42.833	3.492	79.07	0.791	<0.001
	Within populations	20.333	0.924	20.93		
	Total	63.167	4.417			
ITS2	Among populations	27.273	2.455	90.00	0.900	<0.001
	Within populations	5.455	0.273	10.00		
	Total	32.727	2.727			
Combined *cox*1-*cytb*-ITS2	Among populations	113.000	9.983	75.78	0.758	<0.001
	Within populations	63.818	3.191	24.22		
	Total	176.818	13.174			

### 3.9 Neutrality test and demographic history

Neutrality tests based on Tajima’s *D* and Fu’s *F*_*s*_ statistics evaluated the population equilibrium in the two *S*. *calcitrans* populations (Table 6). In the Thai population, the Tajima’s *D* values were consistently negative or close to zero across all markers (-0.217 for *cox*1, -0.123 for *cytb*, -1.430 for ITS2, and -0.158 for the combined dataset), while the Fu’s *F*_*s*_ values were positive (3.907 for *cox*1, 2.180 for *cytb*, 0.506 for ITS2, and 1.269 for the combined dataset). These patterns are indicative of a mutation-drift equilibrium or of weak purifying selection. However, none of the values reached statistical significance, indicating no strong departure from neutrality. In the Spanish population, the Tajima’s *D* (e.g. -1.023 for *cox*1 and -0.567 for the combined dataset) and Fu’s *F*_*s*_ values (e.g. -0.753 and -1.143, respectively) were negative, which may reflect historical bottlenecks or purifying selection. Again, none of these values were statistically significant. For the ITS2 marker, all Spanish specimens shared a single haplotype, rendering neutrality tests inapplicable due to a complete lack of genetic variation.

**Fig. 10:**
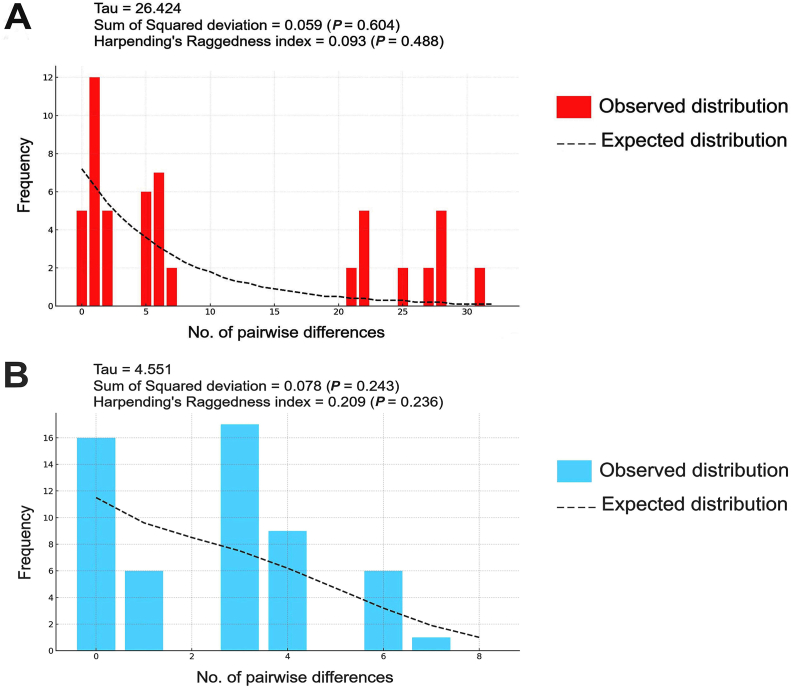
Mismatch distributions of *Stomoxys calcitrans* populations from Thailand (**A**) and Spain (**B**) based on the combined *cox*1-*cytb*-ITS2 sequence dataset. The x-axis represents the number of pairwise differences, while the y-axis indicates their corresponding frequencies. The observed values are depicted as bars, and the dashed line represents the expected distribution under a sudden demographic expansion model. The demographic parameters, tau (τ), sum of squared deviations (SSD), and Harpending’s raggedness index (Rg), are indicated within each panel.

Mismatch distribution analyses were conducted to evaluate the demographic history of *S. calcitrans* populations from Thailand and Spain using the combined *cox*1-*cytb*-ITS2 dataset (Fig. 10). The Thai population exhibited a multimodal mismatch distribution, which deviated from the smooth, unimodal curve predicted under a sudden expansion model. This pattern may reflect a complex population history or underlying population substructure. However, the goodness-of-fit tests did not reject the demographic expansion model, as indicated by non-significant values for both the SSD = 0.059, *P* = 0.604) and Harpending’s raggedness index (Rg = 0.093, *P* = 0.488). The relatively high tau value (τ = 26.424) indicates that any demographic expansion likely occurred in the more distant past. In contrast, the Spanish population showed a distribution pattern more closely resembling a unimodal shape, albeit with minor deviations. This profile aligns more closely with expectations under the recent expansion scenario. Similarly, statistical tests yielded non-significant results (SSD = 0.078, *P* = 0.243; Rg = 0.209, *P* = 0.236), indicating no significant deviation from the sudden expansion model. The lower tau estimate (τ = 4.551) supports the inference that any demographic shift in the Spanish population likely occurred more recently than in the Thai population.

## 4 Discussion

This study builds on previous findings of genetic divergence in global *S. calcitrans* populations to investigate two distinct genetic groups. However, whether this divergence reflects a cryptic species complex or intraspecific variation due to geographical isolation remains unclear. Populations from Thailand (representing the Oriental region) and Spain (representing the Palaearctic region) were compared using wing GM and three genetic markers to assess patterns of divergence and potential species boundaries.

In this study, wing size and shape GM analyses were conducted, each segment revealing statistically significant differences between the two populations. Both male and female *S. calcitrans* specimens from Spain exhibited significantly larger wing CS than those from Thailand. However, wing size alone is not a reliable indicator for species classification, as it frequently overlaps among species and is subject to environmental influences (Dujardin, 2008; Lorenz et al., 2017). Similarly, a previous study investigating *S. calcitrans* populations across Thailand reported significant variations in wing size attributed to differences in local environmental conditions (Chaiphongpachara et al., 2022). Consequently, the wing size differences observed in this study are unlikely to reflect species-level divergence and are more plausibly due to regional environmental variations affecting a single species.

Wing size is typically correlated with overall body size, and larger wings generally indicate a larger body (Outomuro et al., 2021). However, these size differences are likely driven by climatic factors associated with geographical location. Thailand’s proximity to the equator places it within a tropical climate zone, characterized by high humidity and strong monsoonal winds. In contrast, Spain lies within the temperate zone and experiences a Mediterranean climate, marked by lower average temperatures and relatively much colder winters (Arellano et al., 2025). Several entomological studies have found that insect populations inhabiting cooler climates tend to develop larger body sizes than those in warmer regions (Chaiphongpachara and Laojun, 2019; Bota-Sierra et al., 2024; Laojun et al., 2024; Laojun and Chaiphongpachara, 2025). This trend is commonly attributed to slower developmental rates, lower metabolic demands, and improved energy efficiency under cooler conditions (Wonglersak et al., 2020; Bota-Sierra et al., 2024). However, exceptions have been observed, as body size responses may vary depending on species-specific life history traits and local ecological pressures (Abdulloh et al., 2024).

Wing shape analysis, a key variable for species classification using the GM approach, revealed statistically significant differences between the populations of *S. calcitrans* from Thailand and Spain. However, the validated classification based on wing shape demonstrated only moderate discriminatory power with an adjusted overall accuracy of 70%. This contrasts with previous studies that employed similar GM techniques in classifying *Stomoxys* species and reported substantially higher accuracy rates, often exceeding 90% (Changbunjong et al., 2023). To further examine whether the observed wing shape differences were influenced by size, we performed an allometric analysis. The results showed that wing size had a statistically significant effect on wing shape; however, it accounted for only a small proportion of the variation (9.4% overall, 6.6% in males, and 7.9% in females), indicating that while allometry contributes to wing shape variation, its influence is relatively minor. Most of the observed differences in wing shape, including sexual dimorphism, appear to be largely independent of size. Consequently, the moderate classification accuracy likely reflects phenotypic plasticity arising from pronounced environmental differences between the two geographically distant regions, rather than true species-level divergence. These findings suggest that environmental factors play a substantial role in shaping insect wing morphometry. In *S. calcitrans*, for example, larval rearing conditions, such as population density and the type of breeding substrate (e.g. camel, cow, donkey, or sheep dung), significantly affect wing shape (Baleba et al., 2019). Similarly, a study comparing populations from five geographically and ecologically diverse regions of Thailand found significant differences in wing shape across all pairwise comparisons. However, the classification accuracy was relatively low at 54% for females and 61.63% for males (Chaiphongpachara et al., 2022). These findings support the hypothesis that wing morphology variations reflect phenotypic plasticity and local adaptation to environmental conditions (Alves et al., 2016; Yildirim et al., 2024). However, external morphology is influenced not only by environmental factors but also by genetic variation, which is critical in determining morphological traits (Changbunjong et al., 2024). Therefore, the use of GM alone may be insufficient for reliable species-level identification and should be confirmed by molecular data to strengthen taxonomic conclusions.

Molecular analysis-based phylogenetic reconstruction using *cox*1, *cytb*, ITS2, and the combined dataset (*cox*1-*cytb*-ITS2) revealed that *S*. *calcitrans* sequence data, including samples from this study (Thailand and Spain), as well as sequences from European countries retrieved from GenBank, are divided into two distinct lineages: Subclade 1, representing a predominantly European lineage, and Subclade 2, representing a predominantly Thai lineage. Notably, sequences from most East Asian countries, including China and Japan, were grouped within the predominantly European lineage. This result is consistent with previous studies indicating that global *S. calcitrans* populations exhibit distinct genetic structures under two major lineages: the first, found in Oriental populations, and the second, associated with populations from the Afrotropical, Palaearctic (encompassing all European countries as well as northern and central East Asia, including northern and central China and Japan), Nearctic, Neotropical, and Oceanian regions (Dsouli-Aymes et al., 2011). In line with these earlier findings, our study demonstrates that most Thai samples clustered within the Oriental lineage, while the Spanish specimens and European GenBank sequences fell within the second lineage, corresponding to populations from other zoogeographical regions. Despite this phylogenetic structure, all three species delimitation methods used in this study predominantly supported a single OTU for *S. calcitrans*. The only exception was the mPTP analysis of the combined dataset, which indicated the presence of two putative groups, likely reflecting geographical structuring rather than species-level divergence. This interpretation is corroborated by genetic divergence analysis using the K2P model, which identified genetic differences between the Thai and Spanish populations. The highest divergence levels were observed in the mitochondrial genes (*cox*1, 1.99%; *cytb*, 1.51%), which fall below the widely accepted threshold for interspecific differentiation. According to Hebert et al. (2003), interspecific divergence in DNA barcoding studies, particularly for *cox*1, typically exceeds 3%, a benchmark commonly used to delineate species boundaries.

Additional evidence supporting the hypothesis that the two genetic lineages of *S. calcitrans* likely represent a single species is the discovery of a shared haplotype between the Thai and Spanish populations. This observation is consistent with a recent study conducted in central Thailand that identified two distinct genetic clades within Thai populations (Mongkolphan et al., 2023). In the present study, two Thai samples were clustered with the Spanish group in the phylogenetic analysis, one of which shared a haplotype with the Spanish population in the ITS2 region. These findings provide new insights into these two populations, contradicting an earlier study that found no shared haplotypes between Oriental populations and those from other zoogeographical regions (Dsouli-Aymes et al., 2011).

The genetic diversity of *S. calcitrans* from Thailand observed in this study closely resembles that previously reported for the Oriental region, which included Thailand. For example, the *π* and *Hd* for *cox*1 were 0.008 and 0.773, respectively, in this study, compared with the 0.002 and 0.855, respectively, previously reported for the Oriental region (Dsouli-Aymes et al., 2011). Similar trends were found for *cytb* (*π* = 0.006 *vs* 0.004; *Hd* = 0.727 *vs* 0.909) and ITS2 (*π* = 0.001 *vs* 0.004; *Hd* = 0.182 *vs* 0.846). This study represents the first genetic investigation of *S. calcitrans* in Spain; thus no previous genetic data exist for direct comparison. However, relative to other countries in the Palaearctic region (covering Europe as a whole), the Spanish population showed comparable values for *cox*1 and *cytb*, although no variations in ITS2 were observed. Specifically, Spanish *vs* Palaearctic values for *cox*1 were *π* = 0.001 *vs* 0.001 and *Hd* = 0.455 *vs* 0.765; for *cytb*, *π* = 0.001 *vs* 0.004 and *Hd* = 0.485 *vs* 0.507 (Dsouli-Aymes et al., 2011). Across all the genetic markers examined, the Thai population consistently exhibited higher genetic diversity than the Spanish population. Furthermore, AMOVA revealed that most of the genetic variations were due to inter-rather than intra-population differences. These findings indicate that the Thai and Spanish *S. calcitrans* populations possess distinct genetic compositions with clear genetic structuring. Such disparities may be attributed to historical biogeographical and evolutionary processes.

The Oriental lineage, which includes Thai populations, is hypothesized to have diverged from an ancestral group approximately 0.7–1 million years ago during the mid-Pleistocene, followed by subsequent dispersal throughout the Oriental region (Dsouli-Aymes et al., 2011). This prolonged regional presence likely facilitated the long-term persistence and accumulation of genetic variation within geographically isolated populations. This hypothesis is supported by the neutrality test and the mismatch distribution results obtained in this study. For the Thai population, the Tajima’s *D* values were negative or near zero, while the Fu’s *F*_*s*_ values were positive, but none reached statistical significance. These findings indicate no strong recent selection or sudden population expansion. Furthermore, the multimodal mismatch distribution observed in the Thai population indicates a complex demographic history, potentially reflecting the persistent population substructure. In contrast, European populations, such as those in Spain, may have been established more recently than their Oriental counterparts. This more recent establishment, likely involving a limited number of founding individuals, may have had reduced genetic diversity due to founder effects or historical bottlenecks (Dsouli Aymes et al., 2009, 2011). This interpretation is supported by the neutrality test and mismatch distribution results for the Spanish populations. The Tajima’s *D* and Fu’s *F*_*s*_ values were more strongly negative, indicating historical bottlenecks or purifying selection, although none were statistically significant. Furthermore, the mismatch distribution approximated a unimodal pattern, consistent with the recent demographic expansion. The difference in tau values further verifies the hypothesis. The Thai population exhibited a substantially higher tau (26.424) than the Spanish population (4.551), indicating that population expansion in Thailand likely occurred much earlier than that in Spain.

## 5 Conclusions

This study found both genetic and wing morphometric variations between *S. calcitrans* populations from Thailand and Spain, representing the Oriental and Palaearctic regions, respectively. Although wing shape and size differed significantly, these variations are likely due to phenotypic plasticity rather than species-level differentiation. Phylogenetic analyses identified two distinct genetic lineages; however, species delimitation results, low interpopulation genetic divergence, and the presence of shared haplotypes support the conclusion that the two lineages constitute a single, globally distributed species. Neutrality tests and mismatch distribution analyses further indicated that the Oriental lineage has a longer evolutionary history, whereas the European population likely arose from a more recent colonization event. These findings emphasize the significance of historical biogeography in shaping the global genetic structure of *S. calcitrans* and underscore the need for broader geographical sampling to fully elucidate the evolutionary history of the species.

## Ethical approval

The study protocol was approved by the Animal Care and Use Committee of the Faculty of Veterinary Science, Mahidol University, Thailand (Ref. No. MUVS-2024-05-34).

## CRediT authorship contribution statement

**Tanasak Changbunjong:** Conceptualization, Methodology, Formal analysis, Investigation, Writing - original draft, Writing - review & editing, Visualization, Supervision. **Gérard Duvallet:** Conceptualization, Methodology, Investigation, Writing - original draft, Writing - review & editing, Supervision. **Sedthapong Laojun:** Formal analysis, Writing - original draft. **Tanawat Chaiphongpachara:** Conceptualization, Methodology, Formal analysis, Writing - original draft, Writing - review and editing, Visualization, Supervision.

## Funding

This research did not receive any specific grant from funding agencies in the public, commercial, or not-for-profit sectors.

## Declaration of competing interests

The authors declare that they have no known competing financial interests or personal relationships that could have appeared to influence the work reported in this paper.

## Data Availability

The data supporting the conclusions of this article are included within the article. The newly generated sequences were submitted to the GenBank database under the accession numbers PV750979-PV751002 (*cox*1), PV759149-PV759172 (*cytb*), and PV754105-PV754126 (ITS2).
