# Supplementary material for: Is Stomoxys calcitrans a single species? Morphometric and genetic perspectives from populations in Thailand and Spain
Source: Curr Res Parasitol Vector Borne Dis. 2025 Sep 3;8:100315. doi: 10.1016/j.crpvbd.2025.100315 (PMC12452790; doi:10.1016/j.crpvbd.2025.100315)

Supplementary Figure S1. Maximum likelihood phylogenetic tree inferred under the General Time Reversible model with an invariant site distribution (GTR + I), based on *cytb* gene sequences of *Stomoxys calcitrans* from this study (Thailand and Spain; PV759149-PV759172), together with reference sequences from six zoogeographical regions, Oriental, Afrotropical, Palaearctic, Nearctic, Neotropical, and Oceania (indicated by different colored circles), retrieved from GenBank. Outgroup taxa include four additional *Stomoxys* species (*S. bengalensis*, *S. sitiens*, *S. indicus*, and *S. uruma*) and *Haematobosca sanguinolenta*, which lies outside the genus. Bootstrap support values ≥ 80% are indicated at the corresponding nodes. The tree reveals two distinct subclades: Subclade 1, comprising populations from the Afrotropical, Palaearctic, Nearctic, Neotropical, and Oceania regions, and Subclade 2, comprising exclusively Oriental populations. Interestingly, two Thai specimens are grouped within Subclade 1.

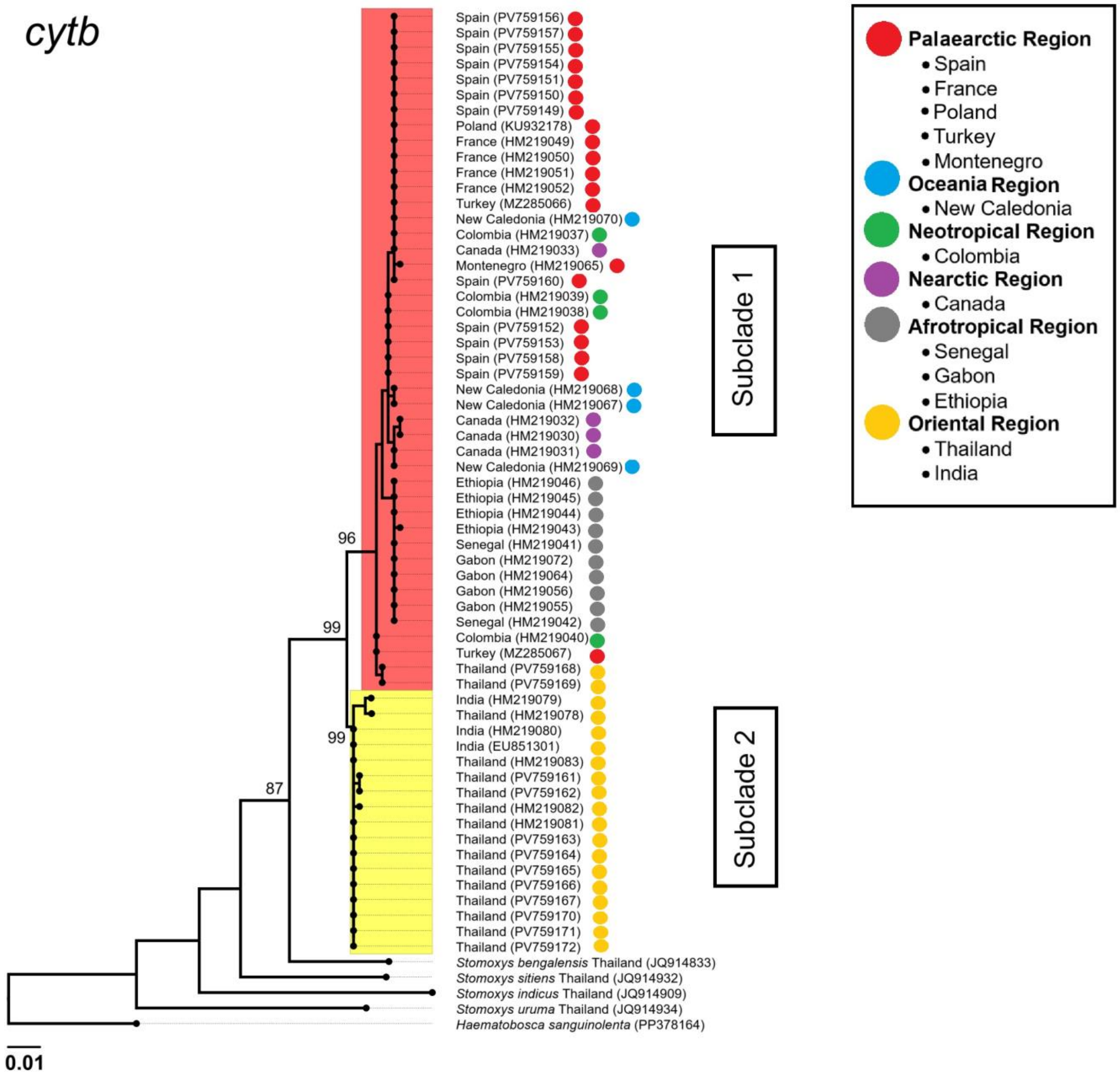

Supplementary Figure S2. Maximum likelihood phylogenetic tree inferred using the Tamura 3-parameter model with Gamma distribution (T92 + G), based on ITS2 sequences of *Stomoxys calcitrans* from this study (Thailand and Spain; PV754105-PV754126), together with reference sequences from six zoogeographical regions, Oriental, Afrotropical, Palaearctic, Nearctic, Neotropical, and Oceania (indicated by different colored circles), retrieved from GenBank. Outgroup taxa include three additional *Stomoxys* species (*S. bengalensis*, *S. indicus*, and *S. sitiens*) and *Haematobosca sanguinolenta*, which lies outside the genus. Bootstrap support values ≥ 50% are indicated at the corresponding nodes. The tree reveals two distinct subclades: Subclade 1, comprising populations from the Afrotropical, Palearctic, Nearctic, Neotropical, and Oceania regions, and Subclade 2, comprising exclusively Oriental populations. Interestingly, two Thai specimens are grouped within Subclade 1.

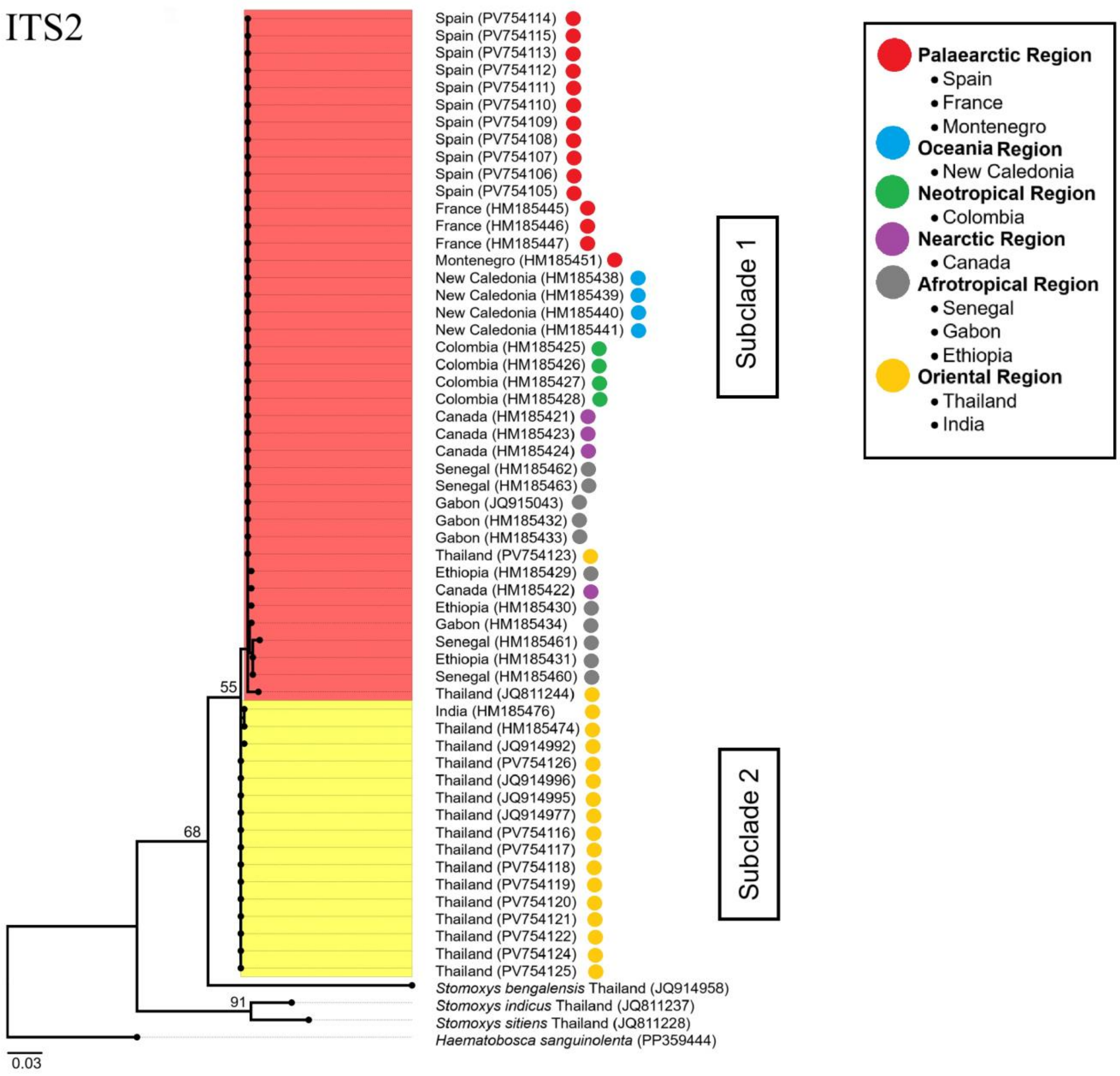

Supplement: Multimedia component 1 [file mmc1.pdf]
